# Supplementary material for: A fault-intrusion-tolerant system and deadline-aware algorithm for scheduling scientific workflow in the cloud
Source: PeerJ Comput Sci. 2021 Nov 2;7:e747. doi: 10.7717/peerj-cs.747 (PMC8576554; doi:10.7717/peerj-cs.747)
Supplement: Supplemental Information 6 [file peerj-cs-07-747-s006.zip › Myproject1.1/docs/org/workflowsim/package-summary.html]

org.workflowsim


JavaScript is disabled on your browser.


- Overview
- Package
- Class
- Use
- Tree
- Deprecated
- Index
- Help

- Prev Package
- Next Package

- Frames
- No Frames

- All Classes

# Package org.workflowsim

- Class Summary

  | Class | Description |
  | --- | --- |
  |  |  |
  | --- | --- |
  | ClusteringEngine | ClusteringEngine is an optional component of WorkflowSim and it merges tasks into jobs |
  | ClusterStorage | ClusterStorage is an extention of HarddriveStorage and it is used as a local storage system of a vm |
  | CondorVM | Condor Vm extends a VM: the difference is it has a locl storage system and it has a state to indicate whether it is busy or not |
  | DatacenterExtended | DatacenterExtended extends Datacenter so as we can use CondorVM and other components |
  | Job | Job is an extention to Task. |
  | Task | Task is an extention to Cloudlet in CloudSim. |
  | WorkflowEngine | WorkflowEngine represents a engine acting on behalf of a user. |
  | WorkflowParser | WorkflowParser parse a DAX into tasks so that WorkflowSim can manage them |
  | WorkflowPlanner | WorkflowPlanner supports dynamic planning. |
  | WorkflowScheduler | WorkflowScheduler represents a scheduler acting on behalf of a user. |
  | WorkflowSimTags | This WorkflowSimTags include tags that are not supported in CloudSimTags |

- Overview
- Package
- Class
- Use
- Tree
- Deprecated
- Index
- Help

- Prev Package
- Next Package

- Frames
- No Frames

- All Classes
